# Irreversible growth plate fusions in children with medulloblastoma treated with a targeted hedgehog pathway inhibitor

**DOI:** 10.18632/oncotarget.20619

**Published:** 2017-09-01

**Authors:** Giles W. Robinson, Sue C. Kaste, Wassim Chemaitilly, Daniel C. Bowers, Stephen Laughton, Amy Smith, Nicholas G. Gottardo, Sonia Partap, Anne Bendel, Karen D. Wright, Brent A. Orr, William C. Warner, Arzu Onar-Thomas, Amar Gajjar

**Affiliations:** ^1^ Department of Oncology, Division of Neuro-Oncology, St. Jude Children’s Research Hospital, Memphis, TN, USA; ^2^ Department of Radiological Sciences, Division of Diagnostic Imaging, St. Jude Children’s Research Hospital, Memphis, TN, USA; ^3^ Department of Pediatric Medicine, Division of Endocrinology, St. Jude Children’s Research Hospital, Memphis, TN, USA; ^4^ Division of Pediatric Hematology and Oncology, University of Texas Southwestern Medical Center, Dallas, TX, USA; ^5^ Starship Blood and Cancer Centre, Starship Children’s Hospital, Auckland, NZ, USA; ^6^ Division of Pediatric Hematology/Oncology, The Haley Center for Children’s Cancer and Blood Disorders at Arnold Palmer Hospital, Orlando, FL, USA; ^7^ Department of Haematology and Oncology, Princess Margaret Hospital for Children, School of Paediatrics and Child Health, Telethon Kids Cancer Centre, Telethon Kids Institute, University of Western Australia, Perth, Western Australia, Australia; ^8^ Department of Neurology, Division of Child Neurology, Lucile Packard Children’s Hospital at Stanford, Stanford University, Palo Alto, CA, USA; ^9^ Cancer and Blood Disorders Program, Children’s Hospitals and Clinics of Minnesota, Minneapolis, MN, USA; ^10^ Pediatric Medical Neuro-Oncology, Dana-Farber Boston Children’s Cancer and Blood Disorders Center, Harvard Medical School, Boston, MA, USA; ^11^ Department of Pathology, St. Jude Children’s Research Hospital, Memphis, TN, USA; ^12^ Department of Orthopedic Surgery, Campbell Clinic, University of Tennessee College of Medicine, Germantown, TN, USA; ^13^ Department of Biostatistics, St. Jude Children’s Research Hospital, Memphis, TN, USA

**Keywords:** premature physeal fusion, medulloblastoma, hedgehog inhibitor, targeted therapy, childhood toxicity

## Abstract

The permanent defects in bone growth observed in preclinical studies of hedgehog (Hh) pathway inhibitors were not substantiated in early phase clinical studies of vismodegib in children. Consequently, vismodegib advanced into pediatric trials for malignancies suspected of being driven by aberrant activation of the Hh pathway. In one multicenter phase II trial, vismodegib was added to the therapy regimen for newly diagnosed Hh pathway activated medulloblastoma. Herein, we report on 3 children (2 on trial and one off trial) treated with vismodegib who developed widespread growth plate fusions that persist long after cessation of therapy. Currently, all 3 patients exhibit profound short stature and disproportionate growth, and 2 subsequently developed precocious puberty. Notably, the growth plate fusions only developed after a prolonged exposure to the drug (> 140 days). These findings resulted in a major trial amendment to restrict the agent to skeletally mature patients as well as a product label warning and update. Moreover, these findings alter the risk-benefit ratio of Hh inhibitors and underscore the importance of careful study of targeted agents in children.

## INTRODUCTION

Medulloblastoma is the most common malignant brain tumor in children. Standard therapy consists of surgery in combination with craniospinal irradiation (CSI) and chemotherapy. Although this treatment cures 60%-70%, survivors of childhood medulloblastoma, experience significant life-long treatment-related morbidities. [1] Neurocognitive impairment, endocrinopathies, and early mortality occur as a consequence of the same broad-based cytotoxic modalities used to combat the disease. [2] Hence, therapy that preferentially targets diseased cells while sparing healthy tissue is eagerly anticipated.

Molecular studies of medulloblastoma show that approximately 25% are Hh pathway activated (hereafter designated as SHH-MB) and inhibitors targeting this pathway, such as vismodegib, sonidegib, and taladegib have been developed for clinical use. [3, 4] Preclinical studies showed these inhibitors were highly potent against mouse models of SHH-MB and had a low toxicity profile in adult mice; [5] however, studies in young mice uncovered severe and permanent defects in bone growth after short (2 day) exposures. [6]

Given these adverse effects in young mice, early phase clinical trials of vismodegib in children included serial monitoring for bone and dental toxicities. Thirty-one skeletally immature children received the Hh pathway inhibitor in phase I (*n* = 23) and phase II (*n* = 8) trials and none had bone or dental toxicities. [7, 8] Moreover, these studies demonstrated anti-tumor activity and prolonged progression-free survival in patients with relapsed SHH-MB. [8]

In 2013, St. Jude Children’s Research Hospital initiated SJMB12 (NCT01878617), a multicenter phase II trial for newly diagnosed medulloblastoma that stratified patients to separate treatment arms by molecular and clinical risk. Vismodegib [dose range 114 - 225 mg per body surface area (m^2^) daily x 28 days x12 cycles] was added after a slightly reduced standardized therapy regimen for SHH-MB patients to evaluate tolerability and study outcomes relative to historical cohorts. This marks the first time that a Hh pathway inhibitor has been used in the up-front treatment setting.

Here, we report 3 patients with SHH-MB treated with vismodegib who developed growth plate fusions. Of them, 2 patients were treated in SJMB12 and 1 patient received single-agent vismodegib off-study after disease relapse.

## PATIENT 1

**Figure 1:**
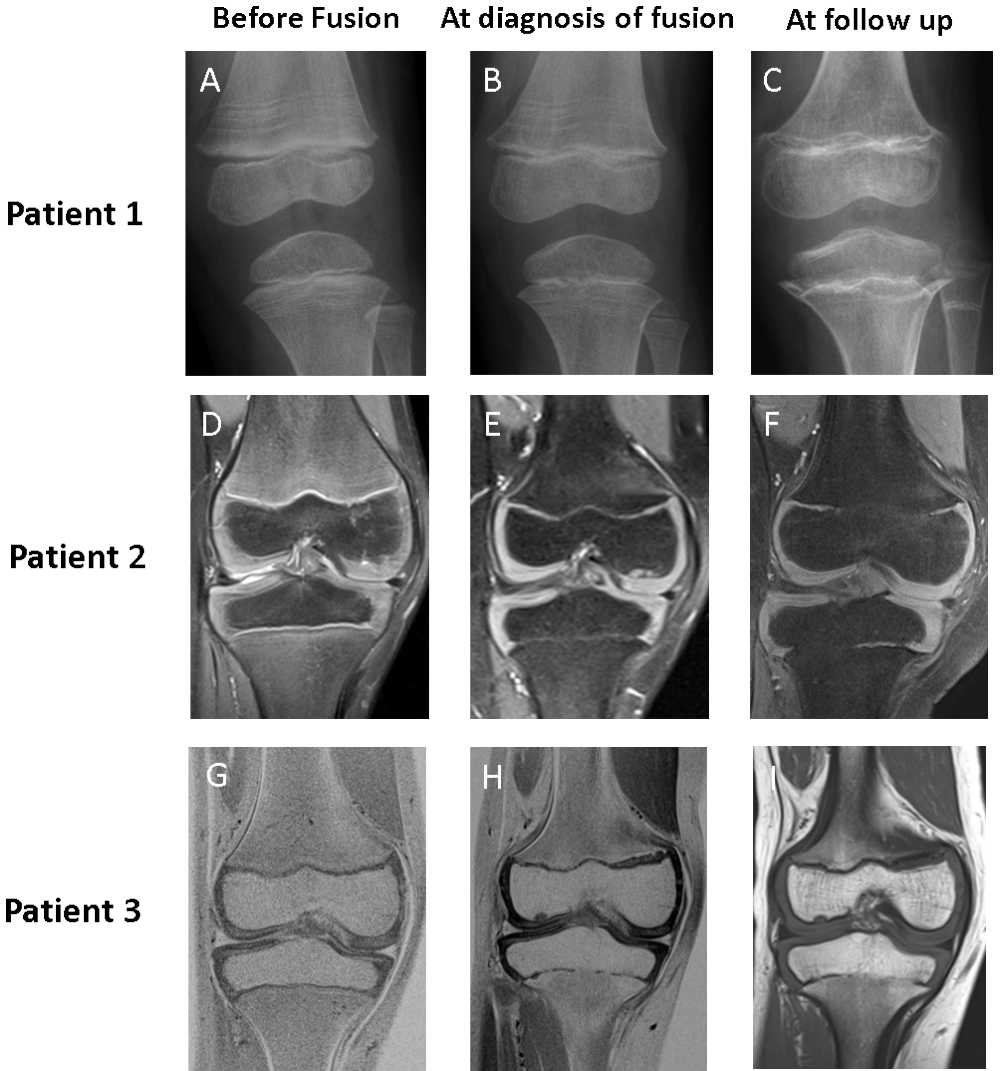
Imaging of knee in patients 1, 2, and 3 before fusion, at diagnosis of fusion, and at follow up Baseline imaging (Panels **A**, **D**, and **G**) show patent physes in all patients at the start of vismodegib therapy. In patient 1, physeal fusions were initially more pronounced in the proximal tibia, incomplete in the distal femur, and absent in the proximal fibula (Panel **B**). Follow-up radiography 17 months after stopping therapy (Panel **C**) showed progression of fusions in all physes and development of abnormal metaphyseal sclerosis. In patients 2 and 3, MRI showed that the initial identification of fusion was subtle, with the development of small bridging fusions at the completion of 12 cycles of vismodegib therapy (Panels **E** and **H**). Over time, these bridges widened to occupy more of the physeal stripe, as revealed by the MRI taken 6 months after the completion of vismodegib therapy (Panels **F** and **I**).

This 5-year-old white female was recently reported to have premature physeal closure after completing 5 cycles of vismodegib. [9] She was diagnosed with SHH-MB at 2 years of age and treated in a St. Jude institutional protocol aimed at reducing radiation exposure in young children with newly diagnosed brain tumors (NCT00602667). At disease relapse, the patient was taken off protocol therapy and the risks and benefits of additional treatment options such as salvage CSI, intravenous chemotherapy, and vismodegib were discussed with her family. Vismodegib therapy was chosen and informed consent obtained. Constrained by the pill strength of 150 mg, the dose approximated 250 mg/m^2^. After 3 months of therapy, magnetic resonance imaging (MRI) showed a near-complete radiographic response and treatment was continued. Toward the end of cycle 5, she complained of bilateral lower extremity pain severe enough to wake her from sleep. A knee X-ray revealed incomplete centralized closure of bilateral proximal tibial and distal femoral physes, which was absent from findings of the knee X-ray taken 4 months earlier (Figure 1). MRI of the brain and spine revealed disease recurrence with leptomeningeal spread. Therefore, vismodegib therapy was discontinued.

Currently, 33 months after the second relapse, the patient continues to battle recurrent disease. Twice she entered remission while receiving cytotoxic chemotherapy, but relapsed within months of stopping therapy. Finally, after her fourth relapse, she underwent CSI and is currently 6 months from completion without evidence of disease.

**Figure 2:**
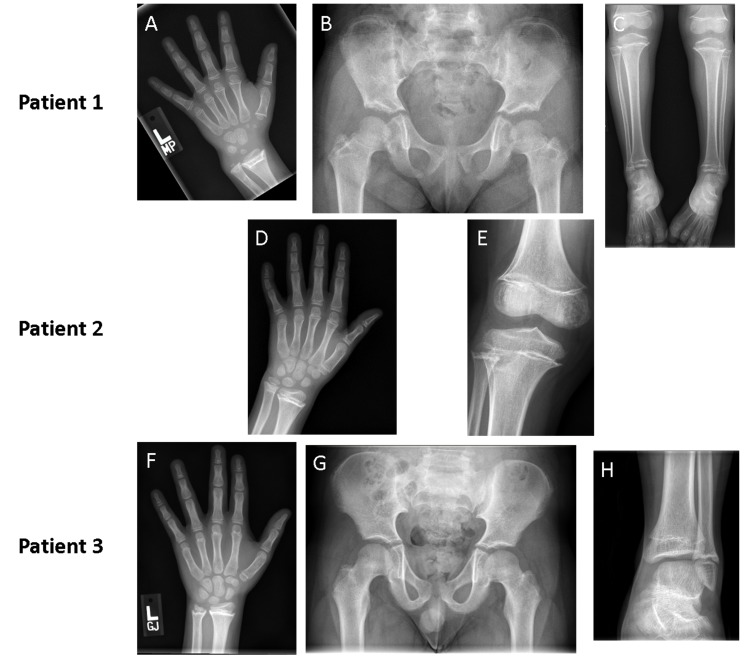
Imaging of patients at follow up showing skeletal deformities resulting from widespread physeal fusions Radiographs of patient 1 at 18 months after cessation of therapy show protrusion of the ulnar head and widespread physeal fusion in hand bones (Panel **A**); abnormally short femoral necks (Panel **B**); and varus ankles, with widespread physeal fusions of bones in the lower leg (Panel **C**). Radiographs of patient 2 at 14 months after completion of vismodegib therapy show widespread physeal fusions in hand and forearm bones (Panel **D**) accompanied by sclerotic thickening in all physes of the knee (Panel **E**). Three radiographs of patient 3 taken 16 months after cessation of therapy show abnormalities that are less prominent but similar to those of patient 1. Advanced bone age (Panel **F**), shortened femoral necks (Panel **G**), and distal fusion of the tibia with relative sparing of the fibula (Panel **H**) was seen.

Over time, effects of physeal fusions have become more evident. Although short stature may in part be attributable to prolonged therapy, her height, which was at the 43rd percentile at 36 months of age, dropped to less than 3rd percentile by 60 months of age (Table 1). Bony protrusions have developed around her wrists, ankles, and knees. Her hands exhibit mild radial deviation around the wrist, with notable projection of the ulnar head. Her ankles demonstrate varus deformity, with the lateral malleolus appearing to overhang the normal position. Prominent fibular heads can be palpated around lateral aspects of her knees. Radiography of bones and joints show widespread systemic physeal fusions with increased metaphyseal sclerosis in phalanges of hands and feet, proximal humeri, distal radii, proximal and distal tibiae, femora, and fibulae (Figure 2). Despite this widespread distribution, all physes are not equally involved and presence of incomplete fusions, alongside complete ones, raise concern that disproportionate bone growth might eventually restrict her ability to walk and use hands normally.

**Table 1:** Patient characteristics at primary diagnosis, 1 year post diagnosis, development of fusion, endocrine visit for fusion, and most recent endocrine visit

Characteristic	Patient 1	Patient 2	Patient 3
Gender	F	F	M
**Characteristics at primary diagnosis**			
Age — y	2.3	4.9	6.7
Pubertal status	Tanner stage 1	Tanner stage 1	Tanner stage 1
Standing height — SD (percentile)	–0.1 (45%)	1.0 (84%)	0.5 (71%)
Sitting height —SD	Missing	0	0.5
Discrepancy in standing to sitting height	Missing	Yes	No
**Characteristics at 1 y post-diagnosis evaluation**			
Age at 1 y post-diagnosis evaluation	3.3	5.8	7.5
Time on vismodegib therapy — mo (cycles)	3 (3)	3 (3)	3 (3)
Pubertal status	Tanner stage 1	Tanner stage 1	Tanner stage 1
Standing height —SD	–0.4 (35%)	0.2 (58%)	0 (48%)
Sitting height –SD	Missing	0	–0.7
Discrepancy in standing to sitting height	Missing	No	No
**Characteristics at development of fusion**			
Age at development of fusion	3.5	6.6	8.2
Time on vismodegib therapy — mo (cycles)	5 (5)	11 (12)	11 (12)
Pubertal status	Tanner stage 1	Tanner stage 1	Tanner stage 1
Standing height — SD (Percentile)	–0.6 (28%)	–0.2 (40%)	–0.6 (29%)
**Characteristics at 1**^st^ **endocrine visit for fusion**			
Age — y	5.1	7.3	8.2
Total time of vismodegib therapy —mo	5	11	11
Time since discontinuation of vismodegib — mo	19	10	0
Pubertal status	Tanner stage 1	Tanner stage 2	Tanner stage 1
Clinical CPP	No	Yes	No
Standing height — SD (percentile)	–1.8 (3%)	–0.7 (23%)	–0.6 (29%)
Sitting height — SD	–1.0	–0.5	–1.0
Discrepancy in standing to sitting height	Yes	No	No
GH peak — ng/mL (*n*>5)	4.7* (failed)	8.6 (passed)	12.9† (passed)
LH baseline – IU/L	0.1	1.88	0.91
Estradiol baseline – pg/mL	0	14	Not applicable
Testosterone – ng/dL	Not applicable	Not applicable	0
LH stimulated – IU/L	Not assessed	Not assessed	12.72†
Testosterone, stimulated – ng/dL	Not assessed	Not applicable	47†
Laboratory results indicating central precocious puberty	No	Yes	Yes†
Bone age — y	5.0	13.5	12.5
Osseous abnormalities in bone age	Yes	Yes	Yes
Treatment	None	GnRHa	GnRHa
**Characteristics at most recent endocrine visit**			
Age — y	5.8	8.8	9.8
Time since discontinuation of vismodegib — mo	28	28	19
Pubertal status	Tanner stage 1	Tanner stage 1	Tanner stage 1
Clinical CPP	Not present	Suppressed	Suppressed
Standing height — SD (percentile)	–2.7 (0%)	–1.4 (7%)	–1.3 (8%)
Sitting height — SD	–2.0	–1.0	Missing
Discrepancy in standing to sitting height	No	No	Missing
Estradiol — pg/ mL	0	0	Not applicable
GH peak — ng/mL (*n*>5)	Not assessed	9.6 ng/mL (Passed)	4.5 ng/mL (failed)
Testosterone — ng/dL	Not applicable	Not applicable	0
Bone age — y	Not assessed	14.5	12.5
Osseous abnormalities in bone age	Not assessed	Yes	Yes
Treatment	None	GnRHa	GnRHa
Counseling	Consider GH replacement		Consider GH replacement.

*GH testing done 6 months later.

†Patient 3 underwent dynamic testing 3 months after initial changes in bone age were observed.

F, female; M, male; y, year; mo, month; SD, standard deviation; CPP, central precocious puberty; GH, growth hormone, LH, luteinizing hormone; GnRHa, gonadotropin- releasing hormone agonist.

Aberrations in Hh pathway genes *PTCH1*, *PTCH2*, and *SUFU* are associated with nevoid basal cell carcinoma syndrome (NBCCS; also known as Gorlin syndrome), which predisposes patients to SHH-MB, basal cell carcinoma, and skeletal abnormalities. However, upon testing, this patient harbored no germline mutations in these genes.

## PATIENT 2

An 8-year-old black female was diagnosed with SHH-MB at 5 years of age. Her medical history was remarkable for congenital bilateral radial and ulnar synostoses and a family history of medulloblastoma in both her father and paternal aunt. Fluorescence *in situ* hybridization (FISH) analysis revealed hemizygous loss of *PTCH1* in tumor cells; however, a *PTCH1* abnormality was not detected upon germline testing. Additional genetic testing to evaluate for other predispositions was offered but declined by the family. Nevertheless, because of high clinical suspicion for NBCCS and resulting heightened sensitivity to ionizing radiation, CSI was omitted from therapy. Consequently, she received SJMB12 protocol therapy with whole posterior fossa radiation instead of CSI, adjuvant chemotherapy, and 12 cycles of vismodegib (188 mg/m^2^/dose). After 6 cycles of vismodegib, MRI of knees showed normal and patent physes. However, after 12 cycles, she had central bony bridging across physes of tibiae, fibulae, and femora. Despite stopping therapy, subsequent imaging revealed progression of central to peripheral closure (Figure 1).

After 10 months, breast budding and pubertal hair growth were noted. Laboratory values confirmed the diagnosis of central precocious puberty (Table 1). Gonadotropin releasing hormone agonist (GnRHa) was given for pubertal suppression to avoid further acceleration of physeal fusions.

Two years after stopping vismodegib, her height has gone from the 83rd percentile at 5 years to the 8th percentile at 8 years. In addition, her bone age has advanced 6 years beyond her chronologic age to 14 years (Table 1 and Figure 2).

## PATIENT 3

A 9-year-old white male was diagnosed at age 6 with desmoplastic/nodular variant medulloblastoma of the SHH subgroup. FISH analysis revealed hemizygous loss of *PTCH1* in tumor cells. He was enrolled in SJMB12 and received 12 cycles of vismodegib (167 mg/m^2^/dose). Screening MRIs of the right knee at start and after 6 cycles of vismodegib revealed normal patent physes. MRI of the same knee after 12 cycles showed early central fusion of the proximal tibial physis. Within 6 months, this physis became progressively and abnormally fused, predominantly in central and medial portions (Figure 1). A partially fused proximal femur physis was also observed with an advanced bone age of 12.5 years.

Although there were no signs of puberty, leuprolide stimulation testing performed 3 months after the bony fusion was consistent with pubertal response, indicating imminent onset of central puberty. Pubertal suppression with GnRHa was initiated for this patient.

Currently, 19 months after stopping vismodegib, his height has gone from the 70th percentile at 6 years to less than the 10th percentile at 9 years (Table 1). Skeletal survey revealed widespread partial and complete physeal fusions similar to those in patient 1 (Figure 2).

## DISCUSSION

The Hh pathway promotes growth, symmetry, and differentiation in various tissues. [10] In mammals, this pathway is activated by the ligands sonic hedgehog (SHH), Indian hedgehog (IHH), and desert hedgehog (DHH). These ligands are specifically expressed in distinct compartments and conservation of these is a mechanism by which developing bodies mediate growth of different tissue types through a single intracellular pathway. [11] SHH primarily activates granule neural cell precursor proliferation in the developing cerebellum, IHH drives chondrocyte proliferation in growth plates of the bone, and DHH is active almost exclusively in gonads. [11] All ligands bind the transmembrane receptor Patched 1 (PTCH1) which triggers its removal from the cilium. This removal of PTCH1 allows the protein smoothened (SMO) to translocate into the cilium where, upon activation, it triggers a signal cascade within the cell. [12, 13]

Current Hh pathway inhibitors function by suppressing SMO, regardless of the Hh ligand. In adults, probably owing to the diminished role of Hh signaling, these agents cause muscle spasms, alopecia, dysgeusia, fatigue, and weight loss. [14] In children, given the pervasiveness of this pathway in developing organ systems, these inhibitors could theoretically have far-reaching systemic consequences.

Indeed, here we describe 3 cases of growth plate fusions in patients on vismodegib and a very recent publication also describes 3 additional cases seen in patients receiving the Hh inhibitor, sonidegib (LDE225). [15] These 6 cases clearly show that disruption of the Hh pathway through SMO antagonism can affect developing humans in a similar manner as developing mice. In mice, exposure to SMO inhibitor resulted in blocked proliferation and early differentiation of chondrocytes, followed by a marked increase in mineralization within the growth plate once the agent was withdrawn; [6] a process that bears eerie resemblance to what has been described in these 3 cases. For the mice, the end result was the shortening of bones and dysplastic joint formation that drew parallels to signs associated with IHH deficiency. [6] Intriguingly, some of the growth abnormalities observed, particularly in Patients 1 and 3 (Figure 2), are also akin to those in patients born with IHH deficiency, which causes the autosomal recessive syndrome acrocapitofemoral dysplasia that is characterized by short-limb dwarfism and brachydactyly. [16]

But how common is this toxicity? Toxic effects related to Hh inhibitors in just 6 patients might not seem substantial given that many children receiving SMO inhibitors have not presented with bone toxicity; however, when duration of exposure and the small number of skeletally immature patients treated are factored in, this number becomes concerning. One important distinction between mouse and man is that bone growth in mice is by comparison extremely rapid and complete at 21 days old. Hence, owing to the much slower growth rate, the time-to-toxicity will predictably be much slower. Indeed the growth plate fusions described here were seen only in patients exposed to 5 or more cycles ( > 140 days), whereas, in early phase clinical trials, median exposure to vismodegib was 2 cycles (56 days). [7] In the phase II study only 1 of 25 received more than 5 cycles of vismodegib and this patient progressed and withdrew from therapy in cycle 6 (162 days). [8] Similarly in the recently published phase I/II trial of sonidegib (LDE225) the 3 pediatric patients who developed growth plate abnormalities discontinued therapy at 57, 169, and 196 days, respectively. By comparison, the median treatment exposure for the pediatric population (*n* = 60) was 55 days suggesting that the vast majority failed to stay on the drug for more than 2 cycles. [15] Consequently, prolonged exposure appears necessary for development of growth deficits and likely explains the paucity of growth plate findings to date.

One major concern is that if the use of SMO inhibitors becomes more commonplace in pediatric oncology then the incidence of this toxicity will incrementally rise. As of February 2016 (when this toxicity was recognized), 23 SHH-MB patients were enrolled in SJMB12. Of them, 9 were skeletally mature (defined as females ≥15 years old and males ≥17). Of the remaining 14 patients, 8 did not receive vismodegib: 3 had not reached this point in therapy, 1 could not swallow tablets, 2 progressed before vismodegib administration, and 2 opted out of study. Of 6 remaining patients, 2 progressed before completing 3 cycles and 4 remained on vismodegib for 5 or more cycles. Two of the 4 (Patient #2 and #3) who completed 12 cycles developed physeal fusion. The other 2 patients, a 9 yr female and a 10 yr old male, completed 6 and 7 cycles, respectively, and had no radiographic evidence of fusion 10 and 17 months from starting vismodegib therapy. Therefore, the actual incidence of this adverse event is currently 50% for skeletally immature patients in SJMB12 who completed 6 cycles and 100% for those who completed 12 cycles of vismodegib.

Even though short stature and growth abnormalities are seen in the pediatric population treated with CSI and intensive adjuvant chemotherapy, the growth abnormalities seen here are notably distinct. Patients receiving multimodality therapy may display short stature from treatment induced growth hormone (GH) deficiency, spinal radiation generating poor sitting heights, and chemotherapy induced reduction in final height. [17-19] However, premature physeal closure is not a finding associated with this multimodal therapy and patient #2 developed a poor standing height despite not receiving CSI, not displaying GH deficiency, and not having a discrepancy in sitting and standing height.

The fact that 2 of the 3 patients with physeal fusion also developed precocious puberty is unexpected. Epiphyseal maturation occurs throughout puberty and is driven by a complex, incompletely understood signaling network involving IHH, parathyroid hormone-related protein, and bone morphogenetic proteins. [20] Consequently, it is possible that a feedback signal from bone to the hypothalamic-pituitary axis was altered due to premature cessation of Hh signaling. Although most interesting, the link between precocious puberty and these growth plate fusions cannot be substantiated on 2 patients alone and it requires a lot more exploration. Nonetheless, since testing is uncomplicated, this observation should elicit surveillance of all patients who experience this toxicity.

Another concern for these patients is that once a growth signal is stimulated, through endogenous GH release or through GH replacement, it may accelerate abnormal growth in more severely affected areas rather than increase the final height. Two patients reported herein have since developed growth hormone (GH) deficiency as expected after CSI exposure [17] and the decision to replace is being debated. Patients have been counseled, and to date none of them opted for GH replacement. Given the anticipated severity of skeletal abnormalities, orthopedics and psychological counsel is being given to all the patients and their families such that an informed decision can be reached.

In conclusion, our observations raise questions about the use of Hh inhibitors in skeletally immature patients. Although responses to Hh inhibitors have sometimes been dramatic, they have been short-lived and restricted to a small subset of SHH-MB patients with defects in the Hh pathway upstream of SMO. [8, 21] Also, responders do not harbor TP53 mutations, which represent a significant subset of SHH-MB with poor prognosis. Therefore, in SJMB12, for newly diagnosed patients with SHH-MB for whom predicted survival after multimodal therapy remains high [3], the putative benefit of Hh inhibitors was not felt to outweigh the risks. Consequently, the protocol was amended to restrict vismodegib to skeletally mature patients. Furthermore, Genentech has updated its product label and issued a warning letter regarding the risk of premature epiphyseal fusion. [22, 23]

In children with incurable medulloblastoma for whom treatment options are limited or absent, the decision to use Hh inhibitors is complex. Tumors should be molecularly vetted against the agent’s mechanism of action to see if these will predictably respond, and, in our opinion, Hh inhibitors should only be used in developing children if the potential benefit is greater than the debilitating risks.

In a broader context, while the introduction of targeted agents heralds an exciting era in medicine, agents that target developmental signaling pathways should be thoroughly evaluated for potential development-specific toxicities. Since pharmaceutical companies and clinical trialists are, correctly, being encouraged to include children in a novel agent’s developmental path, the importance of careful monitoring for toxicities, particularly in the growing and developing child, cannot be overstated.
